# Comparison of Neonatal and Adult Mice-derived Sertoli Cells in Support of Expansion of Mouse Spermatogonial Stem Cells *In vitro*

**Published:** 2012-03-20

**Authors:** Faranak Tavakolifar, Abdolhossein Shahverdi, Mehdi Pirouz, Malak Shakeri, Morteza Koruji, Hossein Baharvand

**Affiliations:** 1 Department of Stem Cells and Developmental Biology, Cell Science Research Center, Royan Institute for Stem Cell Biology and Technology, ACECR, Tehran, Iran; 2 Department of Developmental Biology, University of Science and Culture, ACECR, Tehran, Iran; 3 Department of Embryology, Reproductive Biomedicine Research Center, Royan Institute for Reproductive Biomedicine, ACECR, Tehran, Iran; 4 Department of Animal Science, Agricultural Campus, University of Tehran, Tehran, Iran; 5 Department of Anatomical Sciences, School of Medical Sciences, Tehran University of Medical Sciences, Tehran, Iran

**Keywords:** Spermatogonial Stem Cell, Transplantation, Feeder layer, Sertoli Cell

## Abstract

**Background:**

This study compared neonatal and adult mice-derived Sertoli cells (NSCs and ASCs) to examine the influence of feeder cells derived from donors of different ages on the maintenance of mouse spermatogonial stem cells (SSCs) *in vitro*.

**Materials and Methods:**

SSCs were derived from the testes of six-day-old mice. They were subsequently transferred to Sertoli cells which were isolated by datura stramonium agglutinin (DSA) lectin from neonatal and adult mice for five days.

**Results:**

The numbers of spermatogonial colonies, the numbers of cells per colony, and cloning efficiency were assessed in presence of NSCs and ASCs. The expression of α6- and β1-integrin- positive cells was evaluated. Moreover, the functionality of the cells was assessed by their transplantation into the testes of busulfan-induced infertile mice. Colony efficiency assay showed that the number of colonies derived from single spermatogonial cells were significantly higher on NSCs. Additionally, the transplantation of dissociated colonies into the testes of busulfan-induced infertile mice showed their migration to the seminiferous basal membrane.

**Conclusion:**

These results show that NSCs may provide a more favorable microenvironment in comparison with ASCs for *in vitro* culture of spermatogonial colonies.

## Introduction

A unique population of stem cells in mammalian testes is spermatogonial stem cells (SSCs) that have the capacity for gamete production and transfer of genetic material to subsequent generations (1,2). The preservation of these cells is important for the maintenance of testis tissue and function, and novel options for fertility treatment in oncology patients (3-6). The number of stem cells in the testes is approximately 0.03% of the total testicular cell population in the adult mouse (2, 7). Various methods have been tested in mice with the intent to find an efficient method for the culture and maintenance of SSCs. For example, the purification of SSCs prior to culturing by creating defects in spermatogonial puberty in animals, such as using cryptorchid mice (8-13) and mice that harbor either a c-kit (14) or vitamin A deficiency (15). In addition to purification by fluorescence activated cell sorting (FACS), magnetic activated cell sorting (MACS) of cells that can be marked on the basis of their expression of a cell-type-specific reporter or by cell-surface molecules recognized by antibodies (11, 16) has also been used. Along with an extracellular matrix (ECM) such as gelatin and laminin (1, 2, 16-19) or the administration of various growth factors, (20) the type of feeder cells (STO cells, OP9 bone marrow stroma, L fibroblast cell lines, TM4 or SF7 Sertoli cell lines) (17, 19-22) also influences the isolation and maintenance of SSCs.

SSCs are located close to several supporting somatic cells, including peritubular myoid and Sertoli cells, which may contribute to the formation of an SSC niche. Sertoli cells embrace germ cells of all stages and are joined continuously around the tubule by tight junctions (22, 24). Sertoli cells play important roles in regulation of SSC self-renewal as well as in different stages of spermatogenesis (24-26) as they support SSCs maintenance and proliferation in neonatal mice. Since spermatogenesis begins only after maturation, therefore the ages of the animals (neonatal and adult) may affect the quality of Sertoli cells, as feeder cells, for the maintenance of SSCs.

This study was initiated to compare Sertoli cells derived from neonatal (NSCs) and adult (ASCs) mice with the intent to examine the effect of Sertoli cell age on the properties of mouse SSCs *in vitro*.

## Materials and Methods

### Animals

All animal care and surgical interventions were undertaken in accordance with the approval of the Royan Institutional Review Board and Institutional Ethical Committee. Male mice (NMRI strain, Pasteur Institute, Tehran, Iran) of various ages (neonatal: 6 days; adult: 6-8 weeks) were used to isolate Sertoli cells. Six-day-old mice were used to isolate germ cells. 6-8week-old NMRI mice were used to make busulfan-induced infertile mice.

### Isolation of testicular cells (TCs) and primary culture condition

Isolation of TCs was performed as previously described (29) with slight modification. Briefly, the bilateral testes of 10-15 neonatal mice were collected, placed on ice and then transferred to the laboratory within 15 minutes following decapsulation. Seminiferous tubules were placed in a plate that contained Dulbecco’s Modified Eagle Medium (DMEM; Invitrogen) and separated from one another by pipetting for 5 minutes. The separated tubules were collected by centrifugation at 30×g for 2 minutes, then suspended in DMEM medium which contained enzyme solution that included 1 mg/ml of collagenase/dispase/hyaluronidase and 0.05 mg/ml DNase, for 10-15 minutes (with pipetting) at 37°C. All enzymes were purchased from Sigma-Aldrich. After a second centrifugation, in which the majority of interstitial cells were removed; a second digestion step was performed in DMEM by the addition of fresh enzymes to the seminiferous cord fragments. After filtration through a 40 μm nylon filter, isolated cells were maintained at 32ºC in an atmosphere of 5% CO2 in air for 10 days. The testis cells were cultured in DMEM medium supplemented with 13.5 g/l NaHCO3 (Sigma-Aldrich, USA), single-strength non essential amino acids, 100 IU/ml penicillin, 100 μg/ml streptomycin, and 40 μg/ml gentamycin (all from Invitrogen, USA) with 1% fetal bovine serum (FBS) in the presence of 40 ng/ml of glial-derived neurotrophic factor (GDNF), 20 ng/ml mouse epidermal growth factor (EGF), and 10 ng/ml of human basic fibroblast growth factor (bFGF) (19).The culture medium was changed every three days.

### Preparation of feeder layers

Dissociated testis cells from testes of neonatal mice were allocated to six-well culture plates (1,000,000 cells/9 cm^2^). TCs derived from neonatal and adult mice were isolated by two-step enzymatic digestion with 1 mg/ml of collagenase (30). TCs in DMEM with the above supplements and 10% FBS were placed on datura stramonium agglutinin (DSA, 5 μg/ml, Sigma-Aldrich)-coated dishes and incubated for 1 hour at 37°C in a humidified atmosphere of 5% CO2 in air. After incubation, non-adherent cells were discarded and adherent cells fed by the culture medium for 5-7 days. After 5-10 days, the Sertoli cells formed a confluent layer. STO cells were used as a common feeder layer for culture of SSCs (1, 8, 22, 31). STO cells were cultured in DMEM with the above supplements and 15% FBS for 2-3 days after which their mitotic activation was halted with 10 μg/ml mitomycin.

Germ cells were isolated from the testes of six-day-old neonatal mice and cultured *in vitro* for ten days, then transferred to Sertoli cells isolated by DSA lectin from neonatal and adult mice and STO feeders for an additional five days. All cells were analyzed as following before and after co-culture.

### Analysis of spermatogonial colonies

Analysis of spermatogonial colonies was undertaken in two steps: the first step determined formed colonies ten days after primary culture, and the second step was accomplished five days after transferring the colonies onto three different feeder layers in order to compare the effects of feeder cells on the colonies’ properties.

The number of colonies in each six-well plate was counted by invert microscopy. Surface area of the colonies was measured with Image J software (National Institutes of Health); the number of cells per colony was measured with 100-150 colonies per group.

To evaluate cloning efficiency [(number of colonies/number of seeded cells) × 100], we analyzed the number of colonies of dissociated single spermatogonial cells. The colonies were mechanically separated from culture plates ten days after primary culture of TCs and singled by an enzyme solution that included trypsin-EDTA (0.05%; Invitrogen) and collagenase IV (1 mg/ml). Subsequently, 200,000 cells per group per replicate were seeded and colony efficiency was evaluated during a ten day period.

### RT-PCR analysis

Total RNA was isolated using RNXtm (Cinagene, Tehran, Iran) and treated with a DNaseI, RNase-Free Kit (Fermentas) to remove genomic DNA contamination. One μg of total RNA was used for reverse transcription reaction with the Superscript II Reverse Transcriptase (Invitrogen) and random hexamer primer, according to the manufacturer’s instructions.

The primers used in this study were F: 5' CTT ATC CAA GTT CAC CAG TTC 3', R: 5' TGT ATA AGC CGG AGG TAT 3' for Dazl; F: 5' ACT CCA TTA AAC CAG GAA CCA 3', R: 5' CCC ATT TAA TCT CCT CCT TCT C 3' for Stra-8; and F: 5' GAT AAT CAT TTA GCA CAG CCT C 3', R: 5' GTC AAC AGA TGC AAA CAC AG 3' for mvh (vasa). As a positive control, Gapdh was used.

### Flow cytometry analysis

Single cells were obtained by trypsin from picked up SSC colonies on three different feeder layers and fixed in 4% paraformaldehyde in PBS (pH 7.4) for 20 minutes. Single cells were rinsed with washing buffer (2% FBS in PBS plus 0.029 g EDTA) for 5 minutes prior to blocking in 10% normal goat serum in PBS for 15 minutes, followed by incubation with antibody solution overnight at 4°C. Primary antibodies were rat polyclonal anti-α6-integrin (1:100; Sigma), rat polyclonal anti-β1-integrin (1:100; Sigma-Aldrich) and mouse polyclonal anti-c-kit (1:200; Santa Cruz, CA). The following day, cells were washed twice with washing buffer for 5 minutes and incubated with the appropriate secondary antibody [goat anti-rat and goat anti-mouse labeled with fluorescein isothiocyanate (FITC; 1:200; Sigma-Aldrich)] for 45 minutes, and finally washed twice for 5 minutes before analysis. All steps were performed on ice.

### Immunofluorescence staining

Spermatogonial colonies that were cultured on three different feeder layers and confluent Sertoli cells were fixed in 4% paraformaldehyde in phosphate buffered saline (PBS, pH=7.4) for 20 minutes. Cells were washed twice with 0.1% Tween 20 in PBS prior to blocking in 10% normal goat serum (Vector, Burlingame, CA) in PBS for 15 minutes, followed by incubation with antibody solution against α6 and β1 integrins overnight at 4°C. The following day, cells were washed twice with 0.1% Tween-20 in PBS for 5 minutes, incubated with the appropriate secondary antibody for two hours, and subsequently washed with 0.1% Tween 20 in PBS for 5 minutes. Nuclei were counterstained with 4, 6-diamidino-2-phenylindole (DAPI) in PBS.

### Preparation of recipient mice

To prepare recipient infertile mice testes, busulfan was administered by intraperitoneal injection to mice greater than 4 weeks of age, at a dose of 40 mg/kg, which would almost completely abolish spermatogenesis in most mouse strains (32).

### Preparation of donor cells

Cells for transplantation were obtained from cultured SSC colonies ten days after primary culture and from cultured SSC colonies five days after transfer onto NSCs. Cells were incorporated for 24 hours with BrdU (0.1 mM; Sigma-Aldrich) in culture medium to trace the transplanted cells. Colonies were picked up manually, singled by trypsin and counted. About 100,000 cells per 10 μl medium (plus 5 μl trypan blue to visualize the injection) were used to transplant into each testis.

### Rete-testis micro injection

The abdomens of the recipient mice were opened with a 1.5 cm midline incision. Male mice were placed on the platform and the left testis was exposed. After fixation of the testis, the fat pad of the testes was separated and the position of the rete-testis located. The pipette was inserted into the rete-testis and cells were injected into the tubules. Tubular filling was monitored by observing the movement of the cell suspension which was facilitated by the addition of a small amount of trypan blue to the injection medium. Finally, the skin was sutured.

### Analysis of recipient mice

Recipient males were maintained for 4 to 5 weeks before analysis. Testes were removed and dissected from fat for analysis. The transplanted testes of the recipient mice were fixed in Bouin’s solution for 3-4 days, dehydrated and then embedded in paraffin. The sections were immunostained with a primary anti-BrdU (Sigma-Aldrich) such that donor cell-derived spermatogenesis could be visualized.

### Statistical analysis

The results were expressed as mean±SD. All statistical analyses were conducted using SPSS (version 16) software (SPSS, Inc., Chicago, IL; http://www.spss.com). The statistical significance between the mean values was determined by one-way ANOVA and Duncan’s post-test. P ≤ 0.05 was considered significant.

## Results

**Fig 1:**
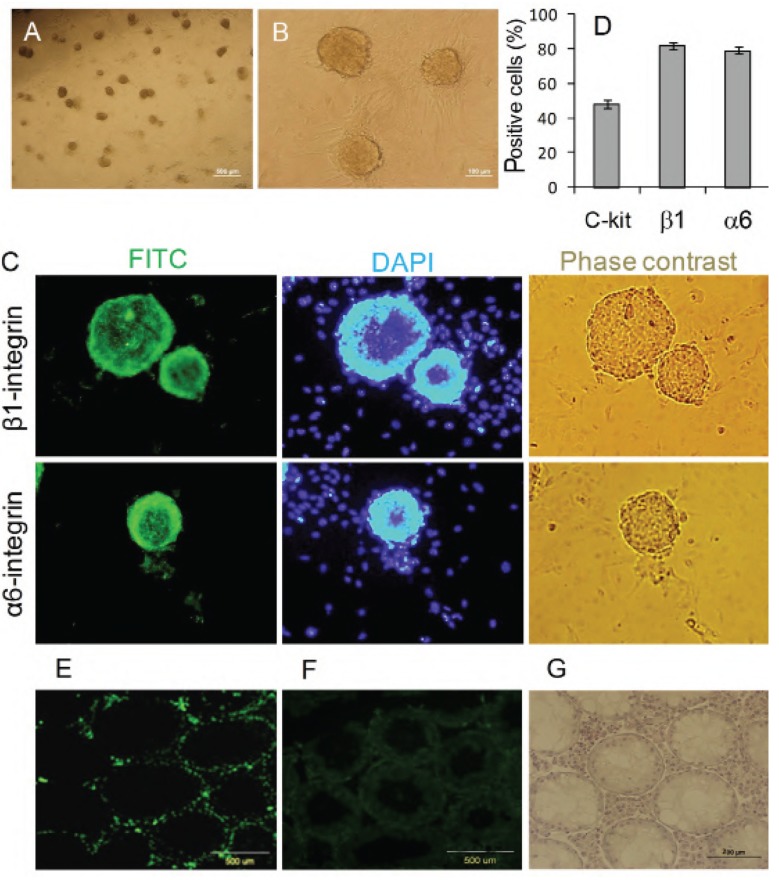
Primary culture of SSC colonies for ten days (A, B). Immunofluorescence staining of spermatogonial colonies for α6-integrin and β1-integrin (C). Flow cytometry analysis of the percentage of a6-integrin-, ß1-integrin-, and c-kit-positive cells (D). Immunohistological tissue section by anti-BrdU shows transplanted BrdU-positive SSCs that are located in the base of seminiferous tubules four weeks after transplantation into the rete-testis of infertile mouse models (E) and the control group (F). H&E staining of testes of infertile mice before transplantation (G).

We cultured 1×10^6^ TC in six-well culture plates for ten days in the presence of 1% FBS, 40 ng/ml GDNF and other growth factors. SSC colonies appeared around three to five days after initial plating of the TCs. These colonies were round; however they contained individually detectable cells (Fig 1A, B). After ten days, the numbers of the colonies were 349.5 ± 58.5 and surface area was 23377.3 ± 1364.5 μm^2^.

Spermatogonial cells were characterized by immunofluorescence and flow cytometry before transferring the colonies to feeder layers (Fig 1C). The colonies were α6-integrin- and β1-integrin-positive which indicated they included SSCs. Flow cytometry analysis of cells ten days after culture showed that the percentages of α6-integrin-positive cells were 79.0 ±1.8%, β1-integrin-positive cells were 81.7 ± 2.0% and c-kit-positive cells were 47.9±2.1% (Fig 1D).

These spermatogonial cells showed the ability to migrate to the seminiferous basal membrane (Fig 1E) after transplantation into the testes of busulfan-induced infertile adult mice, which were depleted of germ cells before transplantation (Fig 1G).

**Fig 2:**
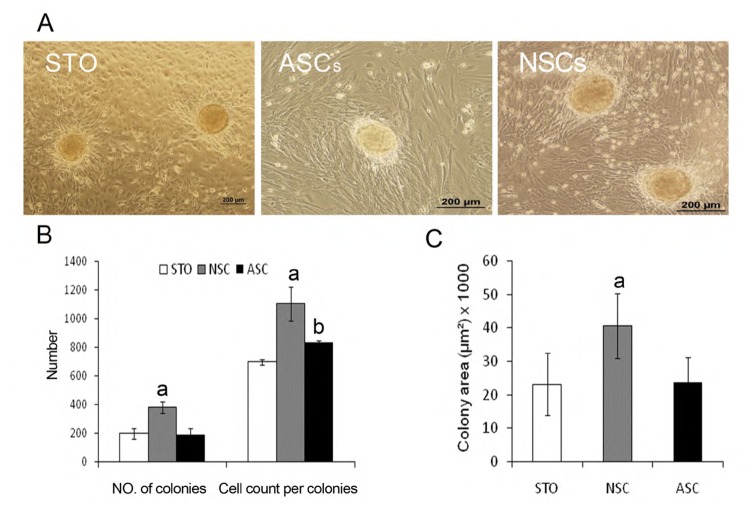
Morphology of SSC colonies five days after culture on STO, ASCs, and NSCs. (A) Colony analysis five days after colonies were transferred to three different feeders. a: NSCs vs. ASCs and STO, p≤0.05. b: ASCs vs. STO, p≤0.05 (B and C).

To evaluate the effect of feeder layer on spermatogonial cell culture, ten days after primary culture of TCs, we manually separated spermatogonial colonies from the culture plates without dissociation and then transferred them to Sertoli cells that were either isolated by DSA lectin from neonatal or adult mice and/or STO feeder cells. The morphological appearance of the colonies five days after transfer to different feeder layers showed that the feeder layers did not affect the shape of SSC colonies. Numerous single cells around surrounded the colonies that were cultured on NSCs, which were absent from the two other groups (Fig 2A).

After transfer to feeder layers for five days; the numbers of colonies, cell counts per colony and surface areas of the colonies were measured. The numbers of colonies on NSCs were 384.3±42.9, ASCs were 193.2 ± 42.5, and STO were 197.8 ± 38.8 (Fig 2B). The cell numbers per colony and surface area of the colonies were 1103.3 ± 118 and 40612.1 ± 5751.9 μm^2^ in the NSCs group; 836.0 ± 158.0 and 23516.2 ± 7777.8 μm^2^ in the ASCs group; 698.3 ± 20.1 and 23250.2 ± 9397.6 μm^2^ in the STO group, respectively. These results revealed that the number of colonies, cell numbers per colony, and surface area of the colonies on NSCs were significantly higher than the two other groups (p<0.05; Fig 2B, C).

**Fig 3:**
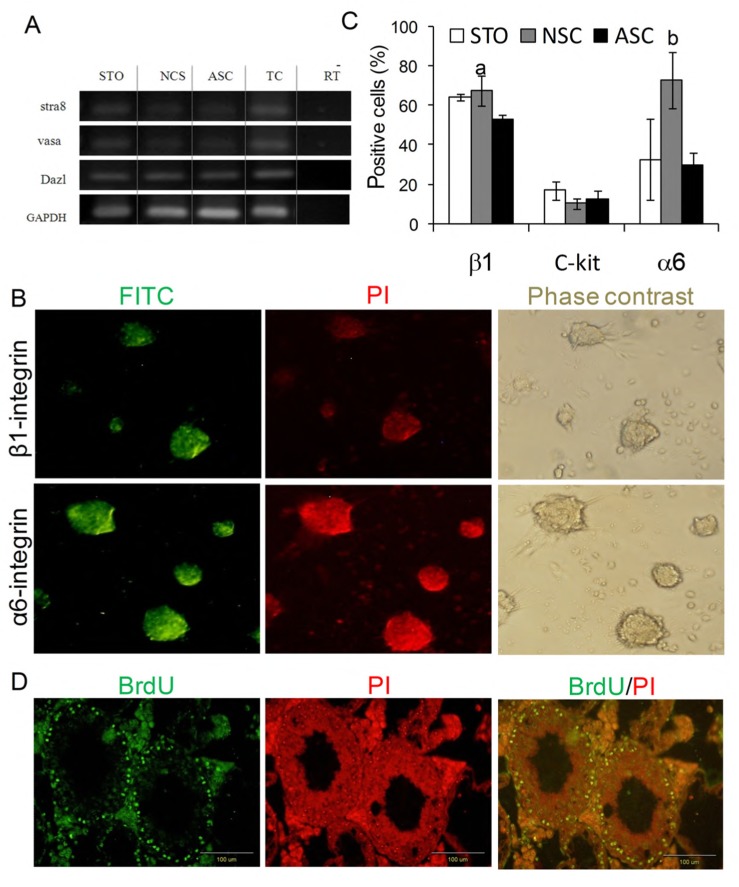
Expression of germ cell markers, including Stra-8, Dazl and Vasa in different groups; TC: testicular cells (A). Immunofluorescence staining of colonies on NSCs by anti-α6-integrin and β1-integrin (B). Flow cytometry results of spermatogonial cell markers (α6-integrin, ß1-integrin and c-kit) for spermatogonial colonies five days after co-culture with three different feeders a: NSC vs. ASC, p<0.05, b: NSC vs. ASC and STO, p<0.05 (C). Transplanted BrdU positive SSCs located in the base of seminiferous tubules four weeks after transplantation into the rete-testis of infertile mice models (D).

Flow cytometry analysis showed a significant increase in the number of α6-integrin-positive cells which were cultured on NSCs (72.9 ± 14.9%) in contrast to those which were cultured on ASCs (29.5 ± 6.5%) and STO (32.5 ± 20.7%); however, there was no significant difference in the number of c-kit-negative colonies (Fig 3C).

RT-PCR analysis was used to evaluate the expression of germ cell markers such as Stra8, Dazl, and Mvh in the colonies of the different groups with TCs as a positive control (Fig 3A).

**Fig 4:**
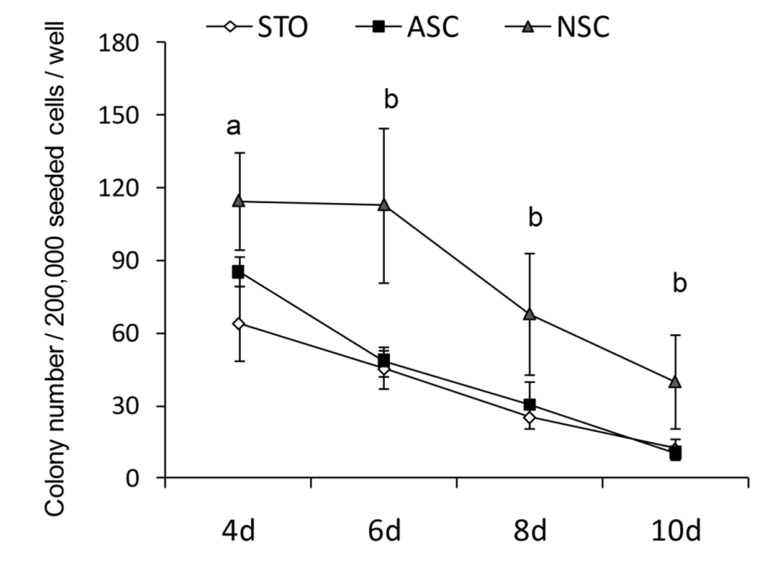
Colony counts from 2x105 cells per well during ten days culture in different groups. The numbers of cultured colonies were: 114.6±20 (day 4), 113±32 (day 6), 68 ± 25 (day 8), and 40 ± 19.4 (day 10) on NSCs; 85.5 ± 6.3 (day 4), 48.5 ± 6.3 (day 6), 30.3 ± 9.8 (day 8), and 10.5 ± 1.7 (day 10) on ASCs; and 64 ± 15.5 (day 4), 45.3 ± 7.8 (day 6), 25.3 ± 4.9 (day 8), and 12.3 ± 4.2 (day 10), a:NSC vs. ASC, p<0.05, b: NSC vs. ASC and STO, p<0.01.

Colony efficiency results showed that the number of colonies during the ten days after culture decreased in all groups (Fig 4).

However, the number of colonies on NSCs was significantly higher in comparison with those on STO (p<0.05) and in comparison with the two other groups (p<0.05). There was no significant difference in the number of colonies at day ten among the different groups. In addition, after transplantation into the testes of busulfan-induced infertile adult mice, the spermatogonial cells cultured on NSCs showed functionality, as verified by their ability to migrate to the seminiferous basal membrane, with no tumor formation (Fig 3D).

## Discussion

In this study, we report the positive influence of NSCs in comparison of STO and ASCs in colony number, cell number per colony, and area of colonies, expression of á6- and â1-positive integrins, and cloning efficiency. These phenomena, in co-culture with NSCs, could be a result of the difference in microenvironments that NSCs and ASCs provide for *in vitro* culture of spermatogonial cells. As spermatogonial cells have been isolated from neonatal mice, another interpretation is that more suitable culture conditions could be obtained when spermatogonial cells and feeder Sertoli cells are derived from mice of the same age.

By considering the biological differences between NSCs and ASCs, such as significantly higher expression of cathepsin L in ASCs when compared to NSCs; the expression of transferrin during maturation and aging of rat Sertoli cells; and increased responsiveness of NSCs to follicle stimulating hormone and decreased responsiveness to androgens (33, 34), one may assume that NSCs could be considered the more suitable feeder cells for neonatal spermatogonial cells. Additional studies are required to shed light on the possible mechanisms involved in the regulation of SSC self-renewal and survival by Sertoli cells. Since Sertoli cells are important in the construction of the SSC microenvironment, future studies would determine whether secreted factors by immature Sertoli cells make them more suitable feeders for SSC culture or the ECMextracellular matrix that is provided primarily by Sertoli cells.
